# Estimating the basic reproduction number of a pathogen in a single host when only a single founder successfully infects

**DOI:** 10.1371/journal.pone.0227127

**Published:** 2020-01-10

**Authors:** Vruj Patel, John L. Spouge

**Affiliations:** National Center for Biotechnology Information, National Library of Medicine, National Institutes of Health, Bethesda, Maryland, United States of America; Universidade Federal Fluminense, BRAZIL

## Abstract

If viruses or other pathogens infect a single host, the outcome of infection may depend on the initial basic reproduction number *R*_0_, the expected number of host cells infected by a single infected cell. This article shows that sometimes, phylogenetic models can estimate the initial *R*_0_, using only sequences sampled from the pathogenic population during its exponential growth or shortly thereafter. When evaluated by simulations mimicking the bursting viral reproduction of HIV and simultaneous sampling of HIV gp120 sequences during early viremia, the estimated *R*_0_ displayed useful accuracies in achievable experimental designs. Estimates of *R*_0_ have several potential applications to investigators interested in the progress of infection in single hosts, including: (1) timing a pathogen’s movement through different microenvironments; (2) timing the change points in a pathogen’s mode of spread (e.g., timing the change from cell-free spread to cell-to-cell spread, or vice versa, in an HIV infection); (3) quantifying the impact different initial microenvironments have on pathogens (e.g., in mucosal challenge with HIV, quantifying the impact that the presence or absence of mucosal infection has on *R*_0_); (4) quantifying subtle changes in infectability in therapeutic trials (either human or animal), even when therapies do not produce total sterilizing immunity; and (5) providing a variable predictive of the clinical efficacy of prophylactic therapies.

## Introduction

When viruses or other pathogens infect a single host, the basic reproduction number *R*_0_ is the expected number of cells infected by a single infected cell [1]. The initial *R*_0_ is a fundamental determinant of whether an infecting viral population will establish itself in the host. On one hand, if *R*_0_ < 1, the viral invaders reproduce below replacement and will go extinct. On the other hand, if *R*_0_ is slightly greater than 1, an initial virus has a small positive probability of amplifying into a systemic infection, and if *R*_0_ is large, infection is all but inevitable.

The initial basic reproduction number *R*_0_ is therefore a continuous variable with direct biological pertinence to infection. As such, it may have many underappreciated applications. As a general example, consider therapeutic trials. More specifically, consider as a motivating application pre-clinical tests of HIV therapies like vaccines in animal models. In this context, the macaque model is popular, because simian immunodeficiency virus can cause a disease progression resembling AIDS in humans [2]. Historically, macaque trials often used a single high-dose intravenous or mucosal inoculation to ensure almost certain infection of unprotected animals [3]. The consequent assessment of therapeutic efficacy depends primarily on binary categorical data, i.e., whether or not infection occurred in treated animals [4,5]. New experimental designs, notably repeated low-dose challenges, have improved statistical power in animal trials [3,6], but do not remove the intrinsic statistical limitations of binary data. The ability to estimate a continuous variable like the initial *R*_0_ in HIV infection would in principle permit a statistically more powerful analysis of therapeutic efficacies.

The initial *R*_0_ is likely a primary determinant of whether systemic infection occurs, and initial microenvironments with relatively few target cells probably impede systemic infection by HIV. Consider, e.g., that the number of per-act transmissions per 10,000 exposures varies considerably by route of infection [7]. For sexual exposure, the number ranges from less than 4 to 138; for needle-sharing, it is about 63. In contrast, for vertical transmission between mother and child, the number is 2260; for blood transfusion, it is 9250. Thus, the initial microenvironment may starkly limit the reproduction of HIV until the virus escapes into systemic circulation, where target cells are plentiful.

Unfortunately, practical thresholds for HIV detection make the initial *R*_0_ inaccessible to direct measurement, because on average, viremia is delayed until about 10 days after exposure to HIV [8,9]. Modern techniques for measuring the abundance of HIV [10] have yielded estimates, e.g., *R*_0_ ≈ 6 [11] or *R*_0_ ≈ 8 [12]. These estimates of *R*_0_ pertain to viremia, however, when there are at least 20 viruses/ml (the current lower limit of detectability) and thus about 10^5^ viruses in the total blood volume of 5L [13]. By the time HIV is detectable in blood samples and *R*_0_ can be measured directly, infection has long since established itself.

The viral dosage may vary considerably between the modes of transmission above, and in sexual or mother-to-child transmission of HIV, e.g., the genetic diversity at early stages of infection usually reflects the number of viruses founding the infection [14–16]. In fact, however, about 80% of all HIV infections arise from a single founding viral sequence [17–19]. Moreover, the design of repeated low-dose challenge animal trials is likely to cause infections with a single founding virus. Thus, because of its practical importance, the case of a single founder virus is a convenient starting point for mathematical analysis, and the rest of this article assumes a single founder.

Although most of this article is self-contained, it continues a scientific program started in [20]. Direct measurement of *R*_0_ early in infection may be impractical, but Fig 1 in [20] showed that the initial *R*_0_ displays its footprint in HIV sequences sampled in early viremia, during exponential expansion of the viral population. To describe the essence of Fig 1 in [20], if only two daughters of the founder successfully contribute descendants to the viremia, and one daughter has a novel mutation away from the founder, about half the sequences sampled in viremia have the mutation. In contrast, if the founder has many daughters successfully contributing descendants to the viremia, far fewer than half the sequences sampled in viremia are likely to have any given mutation.

The structure of this article is as follows: the theory section applies standard statistical procedures to yield a robust method for estimating *R*_0_ from sequence data. The Methods section then describes the simulation of a continuous-time branching process whose parameters are pertinent to sampling sequences of the HIV gp120 gene. The process is the “Gamma model” of [20], a special Bellman-Harris process [21] that idealizes HIV reproduction. The Results section displays the accuracy of our estimator in recovering *R*_0_ in the idealized simulation and the Discussion section examines some consequences of the theory. Finally, the Supporting Information compares our estimator to other (inferior) statistical techniques that we applied to recover *R*_0_ from the same idealized simulation.

All approximations in this article are uncontrolled, i.e., we cannot provide bounds on the error that the approximations cause. Usually without comment, therefore, we rely on simulations of the Gamma model to assess their accuracy. Parameter regimes not pertinent to HIV reproduction and the sampling of gp120 sequences require separate assessment and are beyond the immediate practical purview of this article.

Finally, as motivation, in the context of the Gamma model, our estimator compares favorably with state-of-the-art methods. It has an exceptionally simple analytic form, e.g., yielding a negligible computation time in comparison to exact Bayesian calculations. Moreover, under methods analogous to ours, the coalescent process yields the same estimator as continuous-time branching process models of population growth. Under the Gamma model for HIV reproduction, however, the estimator has a singularity, making it useless for quantitation if *R*_0_ ≥ *e* ≈ 2.7.

## Theory

The following set-up assumes that a single founder virus has infected a single host (e.g., a single HIV infects a human). The set-up is mostly self-contained, drawing only on a few critical approximations presented elsewhere with some mathematical foundations [20]. First, before modeling viral ancestry, we examine the sampling of the viral sequences.

With “≔” denoting a definition, define (*m*) ≔ {1, 2, …, *m*}. Fix an alphabet Λ, e.g., the unambiguous nucleotide alphabet Λ = {*a*, *c*, *g*, *t*}. In practice, sequence analysis must invoke a strategy for handling anomalous characters in an alignment (e.g., ambiguous nucleotides or gap characters). Sometimes, anomalous characters are infrequent, so that as an acceptable approximation, the analysis can treat them as ordinary characters by enlarging its alphabet. Sometimes, the analysis simply omits columns containing them. Without further comment, the following assumes that the practical analysis has adopted an unspecified strategy for handling anomalous characters.

Consider a set *S*_•_ ≔ (*S*_*m*_: *m* ∈ (*M*)) consisting of *M* sequences. The sequences are sampled simultaneously from the descendants of a single founder sequence Φ. Align the sequences *S*_•_, so that the sequences *S*_•_ form an alignment matrix *S*_•,•_ ≔ {*S*_*m*,*n*_: *m* ∈ (*M*), *n* ∈ (*N*)} of *N* columns. Given an unspecified strategy for processing sequences *S*_•_ into *S*_•,•_, the analysis here simply starts with the alignment matrix *S*_•,•_. Implicitly, Φ aligns with *S*_•,•_, so the letter Φ_*n*_ in Φ is ancestral to each letter *S*_*m*,*n*_ in the matrix column *n* ∈ (*N*) (where *S*_*m*,*n*_ is from the sequence *S*_*m*_). In practice, Φ is often unknown, a complication we handle shortly.

The Iverson bracket for indicator random variates is a standard notation [22]: let [*A*] = 1 if the statement *A* is true, and [*A*] = 0 otherwise. Let $M_{n}\left(L\right)≔\sum_{m=1}^{M}\left[S_{m,n}=L\right]$ (*n* ∈ (*N*)) count the instances of letter *L* ∈ Λ in column *n* of *S*_•,•_. Given the strategy for handling anomalous characters, *M* = Σ_(*L*∈Λ)_*M*_*n*_(*L*).

The difference *D*_*n*_ ≔ *D*_*n*_(Φ) ≔ *M* − *M*_*n*_(Φ_*n*_) counts letters in column *n* that have mutated away from the founder Φ; let **D** ≔ **D**(Φ) ≔ (*D*_*n*_(Φ): *n* ∈ (*N*)). Given **D**, let $\eta_{m}≔\eta_{m}\left(\Phi \right)≔\sum_{n=1}^{N}\left[D_{n}\left(\Phi \right)=m\right]$ count the alignment columns *n* ∈ (*N*) where *m* letters differ from the founder letter Φ_*n*_, and define the site frequency spectrum (SFS) as **η** ≔ **η**(Φ) ≔(*η*_*m*_(Φ): *m* ∈(*M*)). Typically, Φ is unknown, so **η** is not observable.

To develop a statistical model for **η**, let *ε*_*n*_ be the probability of a mutation per base per generation in column *n* of the alignment. As in the infinite-sites model [23], we neglect the extremely rare possibility that two or more mutations occur in the ancestry of a single letter *S*_*m*,*n*_. Let $\mu =\sum_{n=1}^{N}\varepsilon_{n}$ be the expected number of novel mutations per generation in the sequences. Despite its biological importance, the effects of preferential selection on sequence data are practically imperceptible during the first six months of HIV infection (see the first paragraph in the Materials and Methods of [19] and Fig 1 in [24]). Assume therefore that (*ε*_*n*_: *n* ∈ (*N*)) are all small, and that novel mutations are all independent. To a good approximation, in every daughter the counts of novel mutations are independent Poisson variates with fixed mean *μ*.

For linguistic convenience, let every virus be both her own ancestor and her own descendant. Let *A*_*m*_ count the non-founder viral ancestors with *m* descendants in the sample, and define as in [20] the ancestral sample frequency spectrum (AFS), **A** ≔ (*A*_*m*_: *m* ∈(*M*)). (Each sampled sequence contributes to *A*_1_, e.g., because by convention, each sampled sequence is its only descendant in the sample). Under an infinite-sites model, every novel mutation occurs in a different column of the alignment. Every alignment column with *m* mutations therefore corresponds to a novel mutation in an ancestor with *m* descendants in the sample [25]. Given **A**, the coordinates of **η** are independent Poisson variates, with *η*_*m*_ having mean *μA*_*m*_ (see, e.g., Theorem 1 in [20]). Accordingly, the relationship is written as **η**=_*d*_ Poission(*μ***A**), where “=_*d*_” indicates equality of distributions.

Eq (17) in [20] used the law of total variance to write

$$\sigma^{2}\left(\eta_{m}\right)=E\left[\sigma^{2}\left(\eta_{m}|A\right)\right]+\sigma^{2}\left(E\left[\eta_{m}|A\right]\right)=E\left[\mu A_{m}\right]+\sigma^{2}\left(\mu A_{m}\right)=\mu EA_{m}+\mu^{2}\sigma^{2}\left(A_{m}\right).$$ (1)

Now, we restrict the discourse to the Gamma model for HIV gp120 (as detailed in the Methods section, which need not be read yet). Simulations of the Gamma model showed [20] that for *μ* = 0.0551 (the value for HIV gp120), the typical magnitude of the ratio $\mu^{2}\sigma^{2}\left(A_{m}\right)/\left(\mu EA_{m}\right)$ from Eq (1) was at most about 18%. For *μ* = 0.0551 in the Gamma model, therefore, the mutational variance $\mu EA_{m}$ makes the dominant contribution to *σ*^2^(*η*_*m*_). The form of Eq (1) shows that the dominance remains robust to varying *μ* (particularly decreasing it), as long as the ratio $\mu^{2}\sigma^{2}\left(A_{m}\right)/\left(\mu EA_{m}\right)$ remains small (say, less than 50%, occurring about *μ* ≈ 0.0551 × (0.50/0.18) ≈ 0.153).

Let $a_{m}≔EA_{m}$ and **a** ≔ (*a*_*m*_: *m* ∈ (*M*)). Given the distributional equality **η**=_*d*_ Poission(*μ***A**), our observations on Eq (1) therefore suggest that the distributional approximation **η**≈_*d*_ Poission(*μ***a**) pertains, as follows. In the present context (the distribution of **η** under *μ* = 0.0551 in the Gamma model), the variation of **A** contributes little to the variance of *η*_*m*_: effectively, as noted by other authors [1,19,24], **A** behaves as though it were the constant $a_{m}=EA_{m}$ when contributing to random fluctuations in *η*_*m*_. In effect, the approximation *A*_*m*_ ≈ *a*_*m*_ treats Eq (1) as an expansion in *μ* around *μ* = 0 and drops terms quadratic in *μ* to retain the approximation $\sigma^{2}\left(\eta_{m}\right)\approx \mu EA_{m}$. As a linear approximation, it should improve as *μ* decreases and worsen as *μ* increases.

To avoid distracting subscripts in the following equations, let *r* ≔ *R*_0_ denote the basic reproduction number *R*_0_ from the Introduction. For some practical purposes, HIV reproduces almost in lockstep, with synchronous generations (see the Delta model of [20], a Galton-Watson branching process [26]). Let *G* count the generations of HIV after host infection. To summarize the previous paragraph,

$$p\left(\eta |G,r,M\right)\approx \prod_{m=1}^{M}e^{-\mu a_{m}}\frac{{\left(\mu a_{m}\right)}^{\eta_{m}}}{\eta_{m}!}.$$ (2)

In any ancestry with *G* synchronous generations, $MG=\sum_{m=1}^{M}mA_{m}$. (Proof: count the ancestors in each of the generations *g* = 1, 2, …, *G*, accounting for the multiplicity *m* of their sampled descendants. The total count is equivalent to counting each of the *M* samples *G* times). Take expectations to derive

$$MG=\sum_{m=1}^{M}ma_{m}.$$ (3)

Reference [20] showed that for the Gamma model,

$$a_{m}^{\left(\Delta \right)}≔a_{m}^{\left(\Delta \right)}\left(r\right)≔\left(\begin{array}{c} M\\ m \end{array}\right)\sum_{g=1}^{\infty }{\left(r^{-g}\right)}^{m-1}{\left(1-r^{-g}\right)}^{M-m}=\sum_{g=1}^{\infty }r^{g}\left(\begin{array}{c} M\\ m \end{array}\right){\left(r^{-g}\right)}^{m}{\left(1-r^{-g}\right)}^{M-m}$$ (4)

accurately approximated $a_{m}=EA_{m}$ (*m* = 2, 3 …, *M*). The heuristic behind the approximation follows. To a good approximation, HIV has synchronous generations *g* = 1, 2, …, *G*. On average, generation *g* contains *r*^*g*^ individuals. Each viral sequence in the sample therefore has an approximate probability *r*^−*g*^ of descending from any particular individual g in generation *g*. Thus, the probability that g has *m* descendants in a sample of size *M* is approximately the binomial probability on the right of Eq (4). Sum the binomial probability over the individuals g in generation *g* (on average, *r*^*g*^ in number) and then over all generations *g* = 1, 2, …, *G*. Let *G* tend to infinity to derive Eq (4).

Eqs (3) and (4) therefore show that if in the Gamma model $a_{1}\approx a_{1}^{\left(\Delta \right)}$, then lim_*G*→∞_(*MG* − *a*_1_) is approximately the (finite) quantity

$$a_{•}^{\left(\Delta \right)}≔a_{•}^{\left(\Delta \right)}\left(r\right)≔\sum_{m=2}^{M}ma_{m}^{\left(\Delta \right)}.$$ (5)

In statistical notations, “•” often suggests a sum, as in $a_{•}^{\left(\Delta \right)}$.

The variable **η** in Eq (2) depends on the unknown founder sequence Φ. To relate **η** to an observable, define ${\tilde{M}}_{n}≔{\text{max}}_{L\in \Lambda }M_{n}\left(L\right)$, the maximum count of any single letter in column *n*. Loosely, ${\tilde{D}}_{n}≔M-{\tilde{M}}_{n}$ then counts minority letters in column *n*. Unlike *D*_*n*_, the observable ${\tilde{D}}_{n}$ has no dependency on the founder sequence Φ. Let ⌊x⌋≔max{i∈ℤ:i≤x} denote the floor function, with $\tilde{M}≔\left\lfloor M/2\right\rfloor$. Define the folded SFS $\tilde{\eta }≔\left({\tilde{\eta }}_{m}:m\in \left(\tilde{M}\right)\right)$ [27], with

$${\tilde{\eta }}_{m}≔\sum_{n=1}^{N}\left[{\tilde{D}}_{n}=m\right]=\sum_{n=1}^{N}\left(\left[D_{n}=m\right]+\left[D_{n}=M-m\right]\right)=\eta_{m}+\eta_{M-m}$$ (6)

for *m* = 1, 2, …, ⌊(*M* − 1)/2⌋), where the second equality holds for an infinite-sites model (which we have assumed). If *M* is odd, $\left\lfloor \left(M-1\right)/2\right\rfloor =\left\lfloor M/2\right\rfloor =\tilde{M}$, so the definition of $\tilde{\eta }$ is complete. If *M* is even, the pattern of pairs *η*_*m*_ + *η*_*M*−*m*_ displayed in Eq (6) fails for $m=\tilde{M}$, because $\eta_{\tilde{M}}$ cannot be paired with a distinct $\eta_{M-\tilde{M}}$. If *M* is even, therefore, define ${\tilde{\eta }}_{\tilde{M}}≔\eta_{\tilde{M}}$ for $m=\tilde{M}$. Loosely, in all cases, ${\tilde{\eta }}_{m}$ counts columns *n* where the number of minority letters equals $m\left(m\in \left(\tilde{M}\right)\right)$.

The folded AFS $\tilde{A}≔\left({\tilde{A}}_{m}:m\in \left(\tilde{M}\right)\right)$ inherits the pattern for $\tilde{\eta }$ established in Eq (6): ${\tilde{A}}_{m}≔A_{m}+A_{M-m}$ for *m* = 1, 2, …, ⌊(*M* − 1)/2⌋; if *M* is even, ${\tilde{A}}_{\tilde{M}}≔A_{\tilde{M}}$. Henceforth and without comment, the same pattern generates folded quantities (denoted by over-tildes) from unfolded quantities, e.g., $\tilde{a}≔E\tilde{A}$. The folded SFS $\tilde{\eta }$ inherits an approximate Poisson distribution from the SFS **η**:

$$p\left(\tilde{\eta }|G,r,M\right)\approx \prod_{m=1}^{\tilde{M}}e^{-\mu {\tilde{a}}_{m}}\frac{{\left(\mu {\tilde{a}}_{m}\right)}^{{\tilde{\eta }}_{m}}}{{\tilde{\eta }}_{m}!}.$$ (7)

Because

$$\underset{G\to \infty }{\text{lim}}\left(MG-{\tilde{a}}_{1}\right)=\underset{G\to \infty }{\text{lim}}\left(MG-a_{1}-a_{M-1}\right)\approx a_{•}^{\left(\Delta \right)}-a_{M-1}^{\left(\Delta \right)},$$ (8)

the pattern suggests imposing the definition

$${\tilde{a}}_{•}^{\left(\Delta \right)}≔a_{•}^{\left(\Delta \right)}-a_{M-1}^{\left(\Delta \right)}.$$ (9)

Eq (7) applied to the observable $\tilde{\eta }$ yields a maximum likelihood estimate (MLE) $\left(\hat{G},\hat{r}\right)$. The asymptotic properties of an MLE make $\hat{r}$ a reasonable benchmark for other statistical estimates of *r*. Recall Eqs (8) and (9) relating ${\tilde{a}}_{1}$ and *MG*, and take natural logarithms in Eq (7) to derive the (approximate) log-likelihood

$$\text{ln}\;L\left(G,r\right)≔\text{ln}\;p\left(\tilde{\eta }|G,r,M\right)\equiv -\mu \left(MG-{\tilde{a}}_{•}\right)+{\tilde{\eta }}_{1}\text{ln}\;\left(MG-{\tilde{a}}_{•}\right)+\sum_{m=2}^{\tilde{M}}\left(-\mu {\tilde{a}}_{m}+{\tilde{\eta }}_{m}\text{ln}\;{\tilde{a}}_{m}\right),$$ (10)

where “≡” indicates an equality of functions, possibly ignoring an irrelevant additive term depending only on data (e.g., a term equaling a function of $\tilde{\eta }$).

As a useful approximation, the following treats *G* as a continuous variate. Now, for any value of *r* (and not just $r=\hat{r}$),

$$\begin{array}{ll} {\text{max}}_{G}\;\text{ln}\;L\left(G,r\right) & ={\text{max}}_{G}\left[-\mu \left(MG-{\tilde{a}}_{•}\right)+{\tilde{\eta }}_{1}\;\text{ln}\;\left(MG-{\tilde{a}}_{•}\right)\right]+\sum_{m=2}^{\tilde{M}}\left(-\mu {\tilde{a}}_{m}+{\tilde{\eta }}_{m}\;\text{ln}\;{\tilde{a}}_{m}\right)\\ & =-{\tilde{\eta }}_{1}+{\tilde{\eta }}_{1}\;\text{ln}\;\left({\tilde{\eta }}_{1}/\mu \right)+\sum_{m=2}^{\tilde{M}}\left(-\mu {\tilde{a}}_{m}+{\tilde{\eta }}_{m}\;\text{ln}\;{\tilde{a}}_{m}\right) \end{array},$$ (11)

where the second equality holds if the maximum is an internal maximum, so the argument $\hat{G}$ is determined by setting the derivative with respect to *G* equal to 0 (i.e., if $\hat{G}$ satisfies $\mu \left(M\hat{G}-{\tilde{a}}_{•}\right)={\tilde{\eta }}_{1}$). An MLE $\hat{r}$ then maximizes the profile log-likelihood, defined as

$$\text{ln}\;L\left(r\right)≔{\text{max}}_{G}\;\text{ln}\;L\left(G,r\right)\equiv \sum_{m=2}^{\tilde{M}}\left[-\mu {\tilde{a}}_{m}\left(r\right)+{\tilde{\eta }}_{m}\;\text{ln}\;{\tilde{a}}_{m}\left(r\right)\right].$$ (12)

Now, ${\tilde{a}}_{m}\left(1\right)={\tilde{a}}_{m}\left(\infty \right)=0$ for $m=2,3,\ldots ,\tilde{M}$. Because ln *L*(*r*) ↓ −∞ at the boundaries of the interval (1, ∞), a MLE $\hat{r}>1$ therefore exists, such that

$$\frac{d}{dr}\text{ln}\;L\left(r\right)=\sum_{m=2}^{\tilde{M}}\left[-\mu {{\tilde{a}}^{\prime }}_{m}\left(r\right)+{\tilde{\eta }}_{m}\frac{{{\tilde{a}}^{\prime }}_{m}\left(r\right)}{{\tilde{a}}_{m}\left(r\right)}\right]=0$$ (13)

has a root at $r=\hat{r}$. In the following, if an MLE lacked an explicit analytic expression, a golden-section search for maxima determined it numerically.

Unfortunately, the direct method of determining $\hat{r}$ by substituting $a_{m}\left(r\right)\approx a_{m}^{\left(\Delta \right)}\left(r\right)$ and then maximizing Eq (12) or solving Eq (13) entails many undependable numerical computations, because Eq (4) for $a_{m}^{\left(\Delta \right)}\left(r\right)$ requires multiplying unreasonably large and small numbers, followed by adding the resulting products with great precision. For completeness, the Supporting Information develops an approximate MLE ${\hat{r}}^{\left(\Delta \right)}$ by approximating *a*_*m*_(*r*) with $a_{m}^{\left(\Delta \right)}\left(r\right)$ and compares it with our best estimator, derived as follows. Approximate the sum in $a_{m}^{\left(\Delta \right)}$ by an integral (the first term of an Euler-Maclaurin series [28]): because (1 − *r*^0^)^*M*−*m*^ = 0 for *m* = 2, 3, …, *M* − 2,

$$\begin{array}{ll} \left(\begin{array}{c} M\\ m \end{array}\right)\sum_{g=1}^{\infty }{\left(r^{-g}\right)}^{m-1}{\left(1-r^{-g}\right)}^{M-m} & \approx \left(\begin{array}{c} M\\ m \end{array}\right)\overset{\infty }{\underset{0}{\int }}{\left(r^{-g}\right)}^{m-1}{\left(1-r^{-g}\right)}^{M-m}dg\\ & =\left(\begin{array}{c} M\\ m \end{array}\right)\overset{1}{\underset{0}{\int }}y^{m-1}{\left(1-y\right)}^{M-m}\frac{dy}{y\;\text{ln}\;r}\\ & =\frac{M}{m\left(m-1\right)}\frac{1}{\text{ln}\;r} \end{array}$$ (14)

where the final equality derives from the evaluation of a beta integral. Comparison of Eq (4) and the two sides of Eq (14) shows that

$$a_{m}^{\left(I\right)}≔\frac{M}{m\left(m-1\right)}\frac{1}{\;\text{ln}\;r}$$ (15)

approximates *a*_*m*_ for *m* = 2, 3, …, *M* − 2.

After substituting $a_{m}^{\left(I\right)}\left(r\right)$ for *a*_*m*_(*r*) in Eq (13) and unfolding all folded quantities,

$$\sum_{m=2}^{M-2}\left[\frac{\mu M}{m\left(m-1\right)}\frac{1}{r{\;\text{ln}}^{2}\;r}-\eta_{m}\frac{1}{r\;\text{ln}\;r}\right]=0.$$ (16)

Telescoping cancellation yields

$$\sum_{m=2}^{M-2}\frac{1}{m\left(m-1\right)}=\sum_{m=2}^{M-2}\left(\frac{1}{m-1}-\frac{1}{m}\right)=1-\frac{1}{M-2}.$$ (17)

To estimate *r*, define ${\tilde{\eta }}_{-1}≔\sum_{m=2}^{M-2}\eta_{m}=\sum_{m=2}^{\tilde{M}}{\tilde{\eta }}_{m}$ (an observable, the sum of all ${\tilde{\eta }}_{m}$ except ${\tilde{\eta }}_{1}$), so the root ${\hat{r}}^{\left(I\right)}$ of Eq (16) satisfies

$$\;\text{ln}\;{\hat{r}}^{\left(I\right)}=\frac{\mu M}{\sum_{m=2}^{M-2}\eta_{m}}\left(1-\frac{1}{M-2}\right)=\frac{\mu M}{{\tilde{\eta }}_{-1}}\left(1-\frac{1}{M-2}\right).$$ (18)

The sum of independent Poisson variates is Poisson distributed, so the variate ${\tilde{\eta }}_{-1}$ is approximately Poisson distributed, with $\sigma^{2}\left({\tilde{\eta }}_{-1}\right)\approx E{\tilde{\eta }}_{-1}$. Define ln^2^ *x* ≔ (ln *x*)^2^. From a linear Taylor series approximation, the approximation $\text{var}\;f\left({\tilde{\eta }}_{-1}\right)\approx {\left[f^{\prime }\left(E{\tilde{\eta }}_{-1}\right)\right]}^{2}\;\text{var}\;{\tilde{\eta }}_{-1}$ with *f*(*η*) = *η*^−1^ yields

$${\hat{\sigma }}^{2}\left(\;\text{ln}\;{\hat{r}}^{\left(I\right)}\right)\approx \frac{{\left[\mu M\left(1-\frac{1}{M-2}\right)\right]}^{2}}{{\left(E{\tilde{\eta }}_{-1}\right)}^{4}}E{\tilde{\eta }}_{-1}\approx \frac{{\;\text{ln}}^{2}\;{\hat{r}}^{\left(I\right)}}{{\tilde{\eta }}_{-1}},$$ (19)

where the final approximation probably has a small relative error if $E{\tilde{\eta }}_{-1}$ is large (i.e., ${\tilde{\eta }}_{-1}\approx E{\tilde{\eta }}_{-1}$).

For comparison of our results with state-of-the-art methods, both the coalescent process and continuous-time branching process models of population growth produce the same approximation, that

$$a_{m}^{\left(C\right)}≔\frac{M}{m\left(m-1\right)}\frac{1}{1-r^{-1}}$$ (20)

approximates *a*_*m*_ for *m* = 2, 3, …, *M* − 2. To relate Eqs (15) and (20), recall the Taylor expansion ln *r* = −ln[1−(1 − *r*^−1^)] ≈ 1 − *r*^−1^ near 1 − *r*^−1^ = 0, i.e., near *r* = 1. The text near Eq (13) of [20] elaborates on the context of Eq (20). For comparison with Eq (18), which gives the estimator ${\hat{r}}^{\left(I\right)}$ in derived from Eq (15), consider the estimator ${\hat{r}}^{\left(C\right)}$ derived analogously from Eq (20),

$$1-{\left({\hat{r}}^{\left(C\right)}\right)}^{-1}=\frac{\mu M}{\sum_{m=2}^{M-2}\eta_{m}}\left(1-\frac{1}{M-2}\right)=\frac{\mu M}{{\tilde{\eta }}_{-1}}\left(1-\frac{1}{M-2}\right).$$ (21)

Routine algebra shows that the estimators are related by the equation

$${\hat{r}}^{\left(C\right)}=\frac{1}{1-\;\text{ln}\;{\hat{r}}^{\left(I\right)}}.$$ (22)

## Methods

Although the biological rationale given below in support of the Gamma model of HIV gp120 is brief, the interested reader can find a more detailed discussion elsewhere [20]. The Gamma model was simulated for each *r* = *R*_0_, as follows. Each realization started with a single successfully infecting founder virus, which (for bookkeeping purposes) died at time *t* = 0, giving birth to a random number *Z*_1_ of successfully infecting daughters. Biologically, each infected cell produces thousands of daughter virions, each with a small independent probability of infecting. The simulation therefore chose the number *Z*_1_ from a Poisson distribution with mean *r* = *R*_0_. Each daughter lived an independent random time after her birth. To approximate life-cycle times relevant to HIV [19,29], the random times had a gamma distribution with mean 2 days and standard deviation 0.24 days. The shape and rate parameters of the gamma distribution were therefore (*n*, *λ*) = (69.4, 34.7) (to make the mean *nλ*^−1^ = 2 and variance *nλ*^−2^ ≈ 0.24^2^). The life-cycle of all the founder’s descendants were similar, making the Gamma model a Bellman-Harris process [21] with parameters specific to HIV.

Each realization generated daughters until there were 6000 live viruses, to mimic a threshold of viral detection in blood. We also examined thresholds larger than 6000, but the exact threshold did not substantially alter scientific conclusions. If the viral population went extinct first, the realization restarted with a new founder. The 6000 live viruses were then sampled to produce six samples, of sizes *M* = 2^*k*^ (5 ≤ *k* ≤ 10). For each sample size *M*, tracing back the ancestry of the sample determined the ancestral sample frequency **A**, which yielded the folded ancestral sample frequency $\tilde{A}$.

In HIV, the gp120 gene is about 2550 nt long, and (with crossovers neglected) each HIV replication averages *ε* ≈ 2.16 × 10^−5^ point mutations/base/replication [1]. On average, therefore, each RNA replication entails *μ* ≈ 0.0551 mutations in gp120. Simulating from the Poisson distribution in Eq (7) with *μ* ≈ 0.0551 yields a folded site-frequency spectrum $\tilde{\eta }$ for the realization.

If ${\tilde{\eta }}_{2}={\tilde{\eta }}_{3}=\ldots ={\tilde{\eta }}_{\tilde{M}}=0$, the realization fails to estimate ln *r*. For each *r* and each *M*, the simulation recorded the number *F* of failed realizations encountered before performing 1000 successful realizations.

For each of the 1000 successful realizations, $\tilde{\eta }$ yielded $\;\text{ln}\;\hat{r}$ for the estimate ${\hat{r}}^{\left(I\right)}$ in Eq (18). The simulation also estimated the corresponding standard deviations $\hat{\sigma }\left(\;\text{ln}\;\hat{r}\right)$ given in Eqs (19). For each *r* and each *M*, the simulation calculated a sample mean $E\left(\;\text{ln}\;\hat{r}\right)$ and sample standard deviation $\sigma \left(\;\text{ln}\;\hat{r}\right)$ from the 1000 successfully sampled values of $\;\text{ln}\;\hat{r}$. It also calculated the sample mean of the estimated standard deviation $E\hat{\sigma }\left(\;\text{ln}\;\hat{r}\right)$ for comparison with the sample standard deviation.

We simulated ancestries as described above for a discrete grid of basic reproduction numbers *r* = (1 + *ε*)^*k*^ (*k* = 1, 2, …,⌊log_1+*ε*_ *R*⌋), so 1 < *r* ≤ *R*, with *R* = 10 and *ε* = 0.1 (as in [20]).

## Results

**Fig 1:**
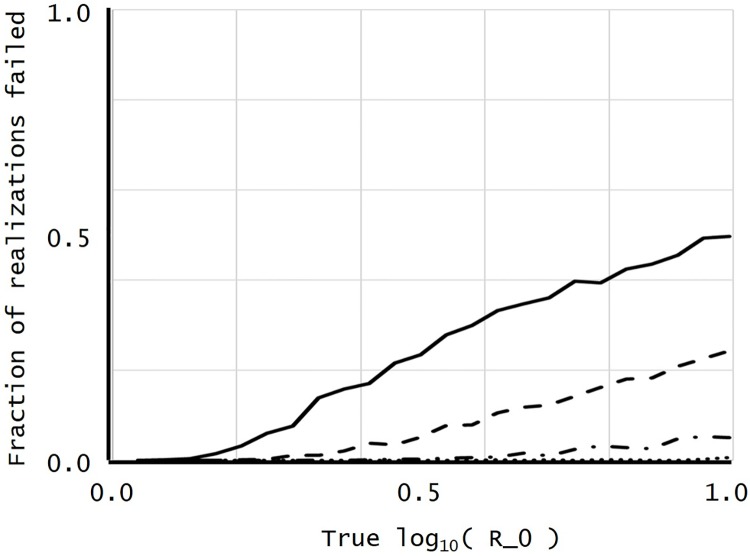
Plot of the fraction of realizations failed against the true log_10_ *r*.

Eq (18) suggests that $\sum_{m=2}^{M-2}\eta_{m}$ tends to decrease as *r* increases (the Introduction refers the reader to Fig 1 in [20] for an intuitive explanation). If $\eta_{-1}=\sum_{m=2}^{M-2}\eta_{m}=0$ in Eq (18), ${\hat{r}}^{\left(I\right)}=\infty$ so one can only infer qualitatively that *r* is large. Thus, if every minority letter in an alignment column is a singleton, making *η*_−1_ = 0, a realization fails to estimate *r* quantitatively. Fig 1 displays the fraction of failed realizations (ones where ${\tilde{\eta }}_{2}={\tilde{\eta }}_{3}=\ldots ={\tilde{\eta }}_{\tilde{M}}=0$).

In Fig 1, the x-axis displays *x* = log_10_ *r* (where *r* = *R*_0_); the y-axis, the fraction of failed realizations. The x-axis runs from log_10_ *r* = 0.0 to log_10_ *r* = 1.0, i.e., *r* = 1 to *r* = 10; the probabilities on the y-axis, from 0.0 to 1.0. The solid line corresponds to *M* = 32; the dashed line, to *M* = 64; the dashed-dot line, to *M* = 128; the dotted line, to *M* = 256. The fraction of failed realizations was identically 0.0 in all simulations with *M* = 512 and *M* = 1024.

**Fig 2:**
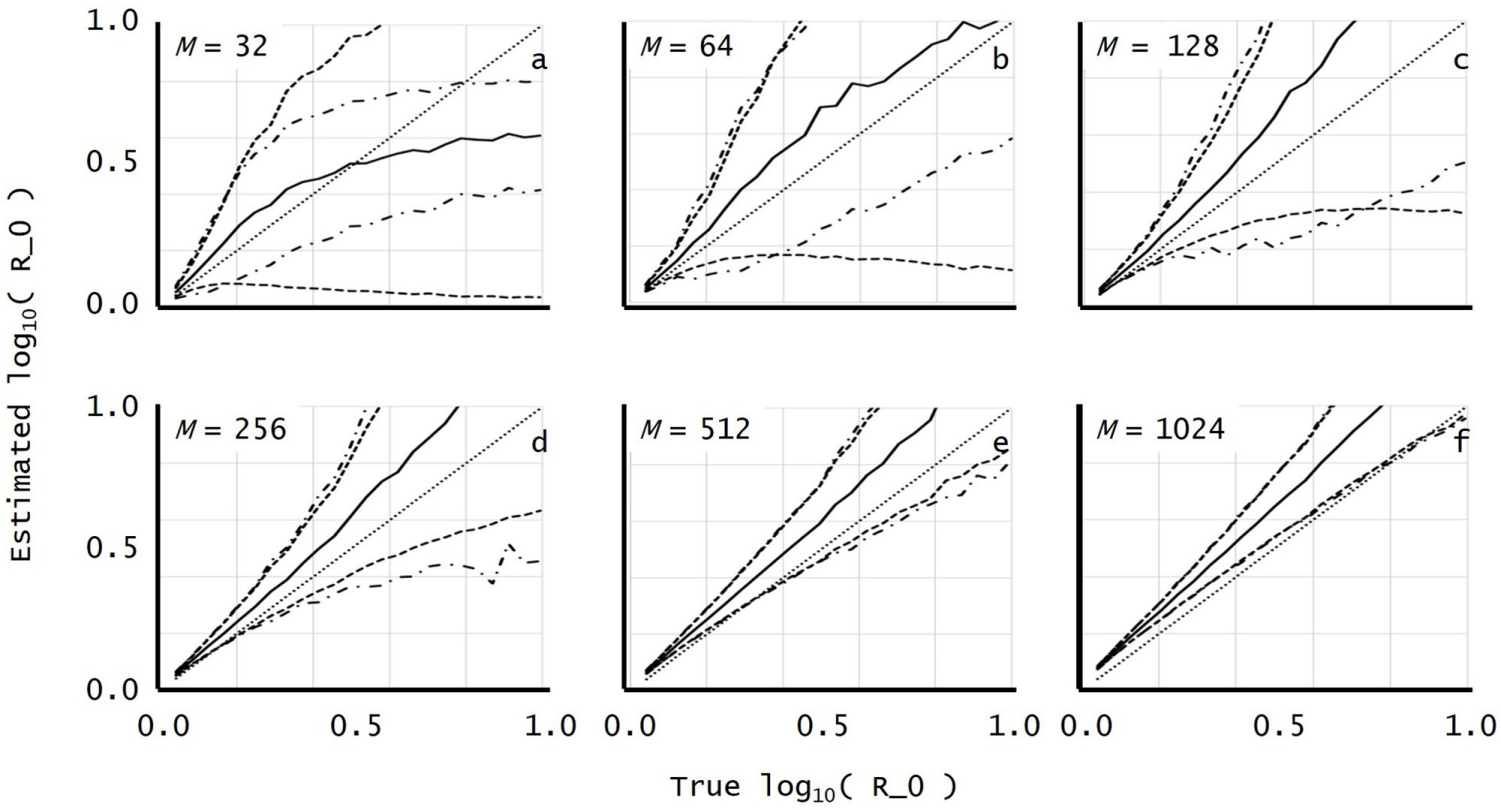
Plots for the maximum likelihood estimate (integral approximation ${\hat{r}}^{\left(I\right)}$).

In each subfigure of Fig 2 for $\hat{r}={\hat{r}}^{\left(I\right)}$ (and also S1 and S2 Figs in the Supporting Information), the solid curves indicate the sample mean $y=E{\;\text{log}}_{10}\;\hat{r}$; the two dashed curves above and below each solid curve indicate $y=E{\;\text{log}}_{10}\;\hat{r}\pm \sigma \left({\;\text{log}}_{10}\;\hat{r}\right)$, i.e., they indicate bands above and below $y=E{\;\text{log}}_{10}\;\hat{r}$ of height equal to the sample standard deviation $\sigma \left({\;\text{log}}_{10}\;\hat{r}\right)$; and the two dot-dashed curves above and below each solid curve indicate $y=E{\;\text{log}}_{10}\;\hat{r}\pm E\hat{\sigma }\left({\text{log}}_{10}\hat{r}\right)$, i.e., they indicate bands above and below $y=E{\;\text{log}}_{10}\;\hat{r}$ of height equal to the sample mean of the estimated standard deviation.

In Fig 2, both x- and y-axes display log_10_ *r*: the horizontal, the true value *x* = log_10_ *r*; the vertical, the estimated value $y={\;\text{log}}_{10}\;\hat{r}$. The x-and y-axes run from log_10_ *r* = 0.0 to log_10_ *r* = 1.0, i.e., *r* = 1 to *r* = 10. The dotted diagonal line indicates perfect estimation, **$\hat{r}=r$**. In their upper left, each of the subfigures (a)-(f) indicates the corresponding sample size *M* = 2^*k*^ (5 ≤ *k* ≤ 10).

Fig 2 for ${\hat{r}}^{\left(I\right)}$ displays progressively better recovery of *r* as M increases. For *M* ≥ 128, the accompanying error estimate ${\hat{\sigma }}^{2}\left(\;\text{ln}\;{\hat{r}}^{\left(I\right)}\right)$ also has practical accuracy. Near log_10_ *r* = 0, Fig 2e and 2f show some systematic overestimation away from the perfect estimate $\hat{r}=r$ (see also S1 and S2 Figs in the Supporting Information).

**Fig 3:**
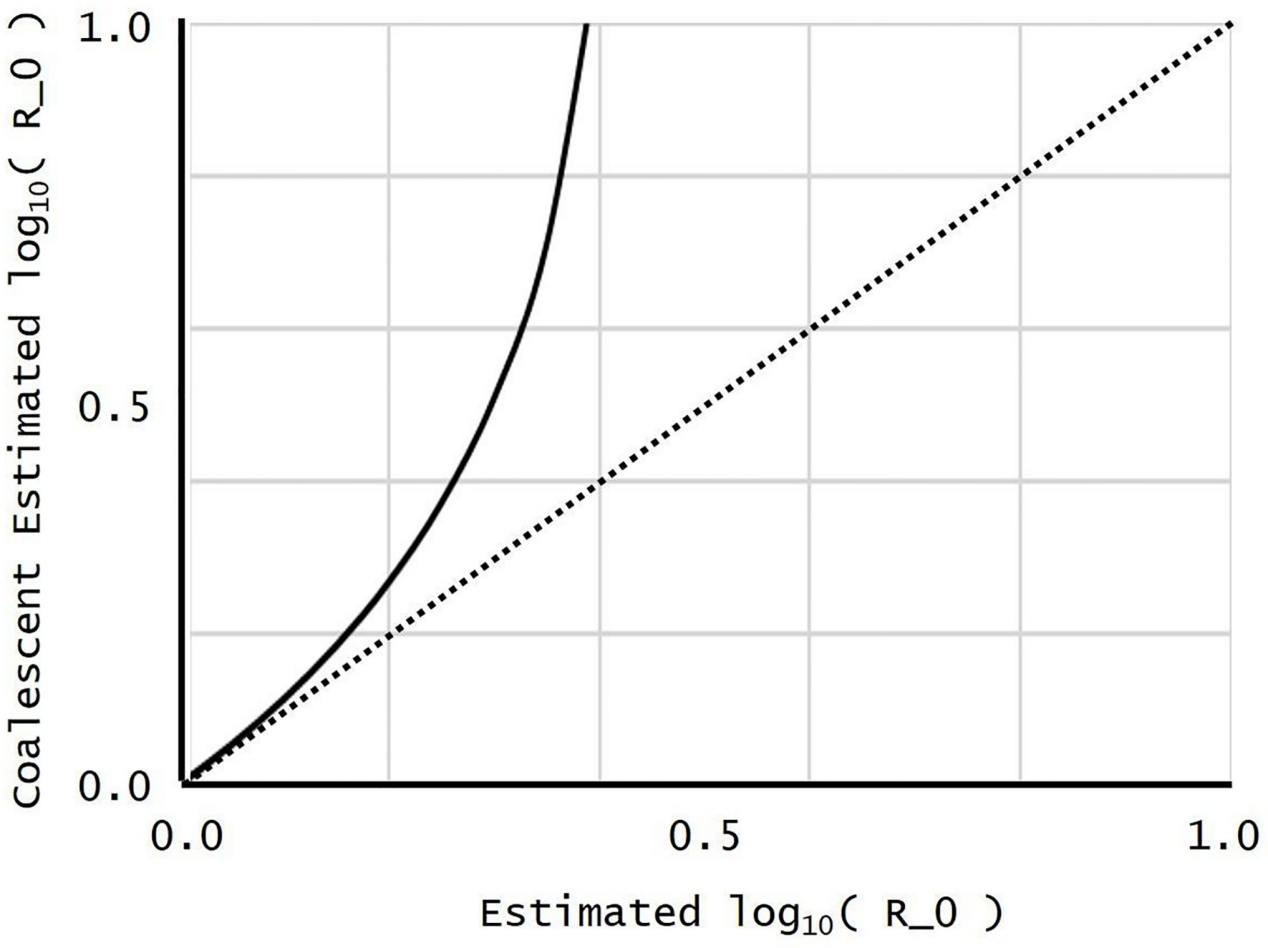
A plot of ${\hat{r}}^{\left(C\right)}$ against ${\hat{r}}^{\left(I\right)}$.

Fig 3 plots $y={\;\text{log}}_{10}\;{\hat{r}}^{\left(C\right)}$ from Eq (22) against $x={\;\text{log}}_{10}\;{\hat{r}}^{\left(I\right)}$. The dotted diagonal line indicates perfect agreement, **${\;\text{log}}_{10}\;{\hat{r}}^{\left(C\right)}={\;\text{log}}_{10}\;{\hat{r}}^{\left(I\right)}$**. In Fig 2, $y={\;\text{log}}_{10}\;{\hat{r}}^{\left(I\right)}$ slightly overestimated the true *x* = log_10_ *r*. In Fig 3, $y={\;\text{log}}_{10}\;{\hat{r}}^{\left(C\right)}$ is consistently larger than $x={\;\text{log}}_{10}\;{\hat{r}}^{\left(I\right)}$. The two estimators ${\hat{r}}^{\left(C\right)}$ and ${\hat{r}}^{\left(I\right)}$ agree well as ${\hat{r}}^{\left(I\right)}$ decreases to 1, in accord with the Taylor expansion near *r* = 1 following Eq (20). (The Appendix of [20] also shows that in the present context, the Delta model of [20], the coalescent model, and the continuous-time branching-process model produce the same limiting SFS as ***r*** decreases to 1). Fig 3 also shows, however, that for larger values of ${\hat{r}}^{\left(I\right)}$, the coalescent estimate ${\hat{r}}^{\left(C\right)}$ becomes a gross overestimate, and it even blows up to infinity at ${\;\text{log}}_{10}\;{\hat{r}}^{\left(I\right)}={\;\text{log}}_{10}\;e\approx 0.434$.

## Discussion

In infection of a single host, the basic reproduction number *R*_0_ is the expected number of cells infected by a single infected cell. This article shows how mutational variations observed in a sample from a population descended from a single founder yield an estimator ${\hat{r}}^{\left(I\right)}$ of *R*_0_. It verifies the accuracy of its uncontrolled approximations with simulations that show that ${\hat{r}}^{\left(I\right)}$ reproduces *R*_0_ within accuracies practicable for some purposes.

Our plots and statistics took log *R*_0_ as the natural scale for the basic reproductive number *R*_0_. The population size at generation *g* is *N*_*g*_ = (*R*_0_)^*g*^, so the population effects of changes in *R*_0_ are linear on a log scale: log *N*_*g*_ = *g* log *R*_0_. On one hand, increasing *R*_0_ by 1 has a greater effect on *N*_*g*_ when *R*_0_ = 1 than when *R*_0_ = 10. On the other, hand, doubling *R*_0_ has the same additive effect on log *N*_*g*_ independent of the value of *R*_0_.

Before considering the biological implications, we make a few technical statistical observations on the estimate itself. In simulations of HIV gp120 sequences, the absence of mutations common to sequence samples can cause estimation of *R*_0_ to fail. For sample sizes *M* ≥ 128, the probability of failing to estimate *R*_0_ was less than 0.051 for all 1 < *R*_0_ < 10 (see Fig 1). To the authors’ knowledge, studies sampling HIV sequences from patients typically sample between *M* = 16 and *M* = 30 [30] sequences per patient, an insufficient depth to test the present theory.

The estimator ${\hat{r}}^{\left(I\right)}$ in by Eq (18) has a simple analytic form, a negligible computation, that should be compared to the complexity of competing Bayesian calculations. Moreover, it does not contain derivatives that may lose accuracy because of numerical differencing. As noted after Eq (18), the coalescent and continuous-time birth-and-death branching process models of population growth yield estimators analogous to ${\hat{r}}^{\left(I\right)}$. In fact, the estimators are the same single estimator ${\hat{r}}^{\left(C\right)}$. The models can be manipulated to yield other estimators, so the comparison of models is by no means exhaustive, but in the present context ${\hat{r}}^{\left(C\right)}$ was clearly inferior to ${\hat{r}}^{\left(I\right)}$. It even displayed a singularity with our simulated HIV data (see Fig 3), suggesting that gradual reproduction in continuous time may have its limits when modeling the lytic viral bursts of HIV.

Like ${\hat{r}}^{\left(C\right)}$, but to a lesser extent, the estimator ${\hat{r}}^{\left(I\right)}$ overestimated *R*_0_ throughout the full range tested, 1 < *R*_0_ < 10 (see Fig 2). Despite the overestimation, which became more severe as *R*_0_ increased, estimates were accurate enough to be practicable for some purposes. As Fig 2 indicates, because of monotonicity of ${\hat{r}}^{\left(I\right)}$, experimental results with a large enough dataset can demonstrate a decrease in *R*_0_. Moreover, near *R*_0_ = 1, both the overestimation and the estimated error appeared relatively small, a useful property for detecting subtle therapeutic progress in reducing *R*_0_. In summary, the integral estimator ${\hat{r}}^{\left(I\right)}$ is easy to compute throughout the full range 1 < *R*_0_ < 10 tested; its bias is noticeable not excessive for all sample sizes *M* ≥ 32; and its error estimates are generally reliable for all sample sizes *M* ≥ 128 (see Fig 2 for details).

The estimator $\;\text{ln}\;{\hat{r}}^{\left(I\right)}$ in Eq (18) is linear in *μ*, suggesting that Eq (18) provides the first term of an expansion that our approximations have linearized around *μ* = 0. Other articles [1,19,24] introduced and justified the linearizing approximation of an unvarying phylogeny, given here after Eq (1). The theory following Eq (1) indicates that the approximation steadily loses accuracy as *μ* increases, but simulations show that it retains enough accuracy to remain practicable in parameters ranges pertinent to HIV gp120.

We now turn to biological considerations. The chief limitation of our study derives from its attempt to use a simple mathematical model capture the complex biology of HIV infection. In fact, *R*_0_ is likely to change as infection reaches new microenvironments in the host. Naïve use of the estimates here can only produce a single effective *R*_0_ for early infection. In future (but beyond the purview of this article), we plan to incorporate time-dependencies into the simulation of *R*_0_, and to develop estimates that can recover some of the time-dependency.

Presently, our mathematical model assumes a single founder. The extension of mathematical modeling from a single founder to multiple founders is an important relaxation of assumptions [19]. Regardless, the single-founder assumption is often satisfied in HIV infection, because most HIV infections have a single founder. For simplicity, it also assumes a constant *R*_0_. After initial infection, HIV traverses different host microenvironments, potentially undergoing genetic bottlenecks. On one hand, a bottleneck can bias estimates based on sequence samples, because they obscure whether a most recent common ancestor dominates early in infection (a founder) or only after the bottleneck. In the present context, therefore, bottlenecks may bias estimates away from an initial *R*_0_ and towards *R*_0_ for a later microenvironment. On the other hand, even multiple escape lineages do not seriously bias genetic estimates of time since infection [24], so estimates of the initial *R*_0_ may share a similar robustness.

Estimates of *R*_0_ may also clarify HIV biology as an infection progresses. If target cells are scarce in a microenvironment, HIV may proliferate predominantly by cell-free spread, budding from an infected target cell, entering the extracellular fluid, and infecting another target cell by chance encounter [31]. Conversely, if target cell are plentiful, e.g., in the microenvironment of a lymph node [32,33], direct cell-to-cell spread may be more efficient than cell-free spread. Cell-to-cell spread has two distinct mechanisms (and therefore can occur in qualitatively distinct environments): (1) transmission of HIV by virological synapses between adjacent target cells or (2) transmission by capture and transfer of virions between proximal macrophages and dendritic cells [31]. Viral replication may not occur during cell-to-cell transmission, so regardless of the exact mechanism, shifts between cell-free spread and cell-to-cell spread may manifest themselves as concomitant changes in *R*_0_. However fundamental *R*_0_ may be to describing the reproduction of pathogens, cell-to-cell spread exemplifies the difficulties in interpreting an estimate of *R*_0_ biologically.

The route of infection determines the initial microenvironment of HIV. Most routes transmit HIV much less effectively than hematological routes [7], suggesting that their initial *R*_0_ is typically low. Mucosal infection can promote transmission of HIV [34], however, because it can increase the local concentration of activated T cells, promoting cell-to-cell spread, and probably increasing the initial *R*_0_. Thus, the initial *R*_0_ may provide valuable information about initial infection.

The dominance of cell-to-cell spread over cell-free spread may vary during infection. After infecting in a cell-rich mucosal microenvironment, HIV may move through the mucosal lamina, before being transported through the lymphatic system to lymph nodes, where the target cell density in its microenvironment increases dramatically [32,33]. The values of *R*_0_ probably vary accordingly.

The present theory may also have an important application in animal trials of viral prophylaxis, when progress towards a therapy is subtle. Indeed, the design of animal trials using high-dose challenges may have unintentionally impeded practical assessment of candidate HIV therapies, because some vaccines and prophylactics may mitigate low- but not high-dose challenges [6]. Repeated low-dose challenge studies represent an important step forward in the pre-clinical assessment, because they mimic typical HIV challenges in humans [3]. Repeated low-dose challenges probably yield infections with a single founder virus, satisfying the primary assumption of the present theory. An estimate of *R*_0_ in this context provides a new variable for statistical analysis, beyond the binary infection status of an animal, one with direct biological relevance to the establishment of infection. Even if a vaccine or prophylactic fails to produce total sterilizing immunity, a reduction in the initial *R*_0_ encourages further investigation of an intervention, where previously the entire line of research might have been discarded.

Trials using repeated low-dose challenges also pose some unanswered experimental questions, the most pressing being the possibility that unsuccessful challenges potentially perturb the challenged animals. Do unsuccessful challenges foster partial immunity to further challenge? Do they increase the probability of future infection? Subtle perturbations in *R*_0_ may be much more sensitive than a binary infection status in providing the answers to such questions.

Next-generation deep sequencing can produce around 10^5^ reads of size comparable to gp120 [35,36]. Given the expectation that future experiments will likely be able to generate datasets with potentially even greater than 10^5^ sequences, any robust estimator of *R*_0_ must be able to handle extremely large sample sizes efficiently. The consistent tightening of the error estimates and accuracy as *M* is increased in Eq (19) suggests that the estimator ${\hat{r}}^{\left(I\right)}$ is particularly well-suited to application in such experiments. In addition, although a complete likelihood for the entire phylogeny and mutations might be desirable in some circumstances, a maximum likelihood method for the SFS permits inference about *R*_0_ even if the reads are short, if the reads can be placed against a reference HIV genome.

In clinical trials of HIV therapies, guidelines previously suggested starting treatment only when CD4^+^ T cell density declines below 350 cells/μl [37]. Recent studies (notably the SPARTAC trial) of temporally earlier interventions have shown increased efficacy compared to standard anti-retroviral protocols [38,39]. In some circumstances, the initial *R*_0_ might measure the clinical efficacy of some such interventions, provide a variable predictive of their efficacy, or even help predict the clinical intervention with the greatest chance of success.

In conclusion, in the case of HIV and possibly other infectious agents, the integral approximation ${\hat{r}}^{\left(I\right)}$ provides a simple, easily computed estimate of the early basic reproduction number *R*_0_ in a single host. The quantitative variable *R*_0_ makes a well-characterized biological contribution to early HIV infection and should be useful assessing the efficacy of therapies in both human and animal trials.

## Supporting information

S1 FigPlots for the maximum likelihood estimate (delta approximation) ${\hat{r}}^{\left(\Delta \right)}$.(TIF)

S2 FigPlots for the estimate ${\hat{r}}^{\left(M\right)}$ from the method of moments.(TIF)

S1 Data (DOCX)
