# Supplementary figures and images for: Estimating the basic reproduction number of a pathogen in a single host when only a single founder successfully infects
Source: PLoS One. 2020 Jan 10;15(1):e0227127. doi: 10.1371/journal.pone.0227127 (PMC6953795; doi:10.1371/journal.pone.0227127)

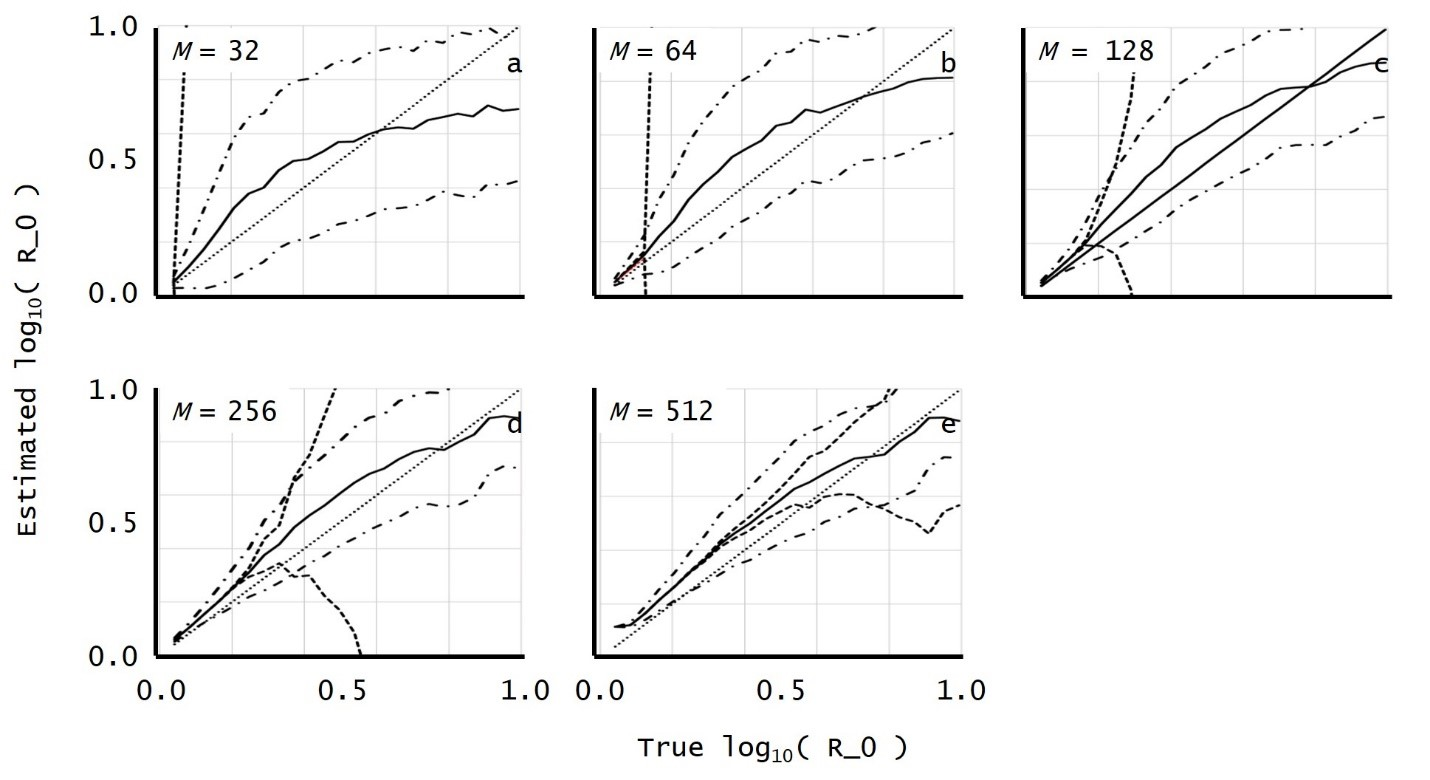

Supplement: S1 Fig — (TIF) [file pone.0227127.s001.tif]

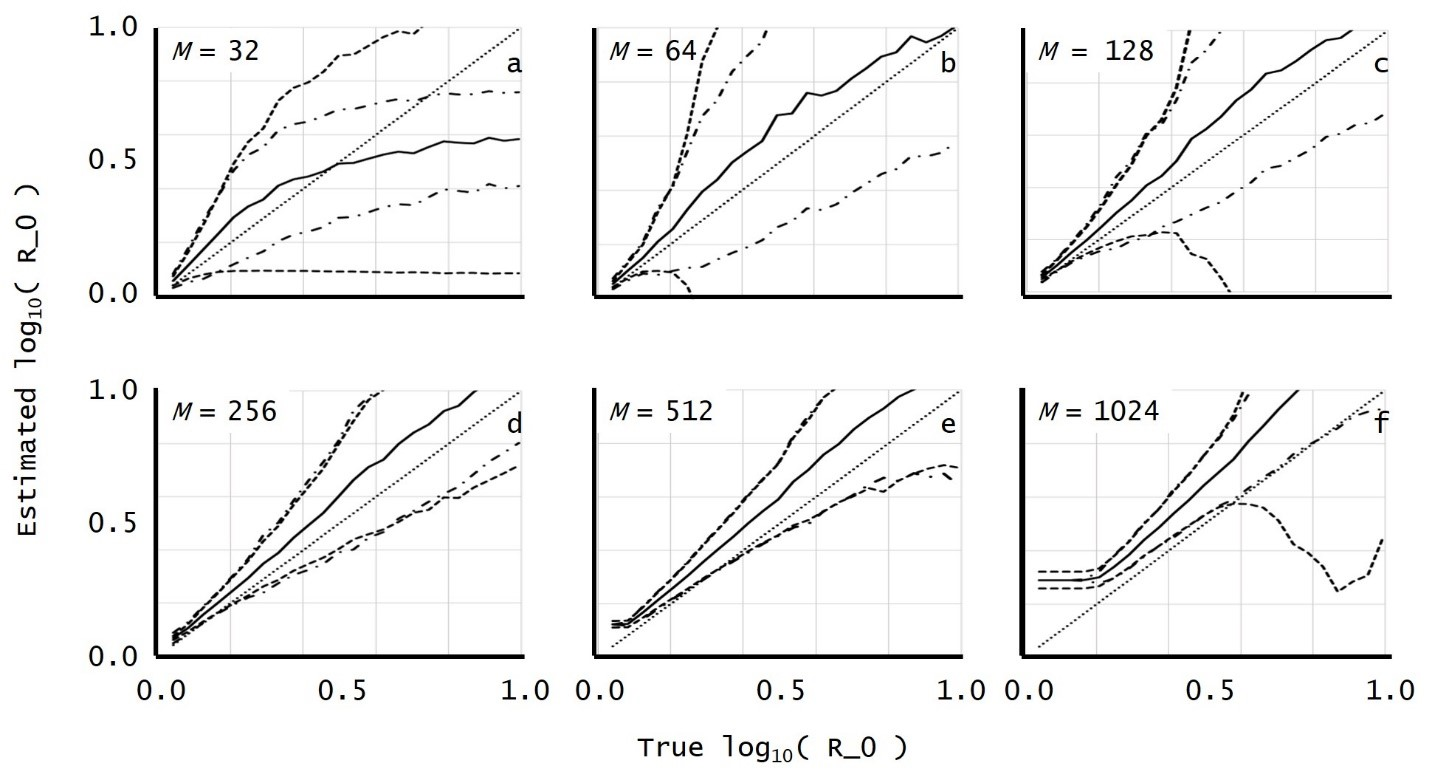

Supplement: S2 Fig — (TIF) [file pone.0227127.s002.tif]
