# Supplementary material for: Estimating the basic reproduction number of a pathogen in a single host when only a single founder successfully infects
Source: PLoS One. 2020 Jan 10;15(1):e0227127. doi: 10.1371/journal.pone.0227127 (PMC6953795; doi:10.1371/journal.pone.0227127)
Supplement: S1 Data — (DOCX) [file pone.0227127.s003.docx]

# Supporting Information

In this section, standard statistical procedures yield two estimators for , $\hat{r}^{\left(\Delta\right)}$ and $\hat{r}^{\left(M\right)}$. They do not appear in the main text, because they prove inferior to $\hat{r}^{\left(I\right)}$.

First, after substituting $a_{m}^{\left(\Delta\right)}\left(r\right)$ for $a_{m}\left(r\right)$, Eq (12) can be maximized numerically to generate a MLE estimator $\hat{r}=\hat{r}^{\left(\Delta\right)}$. Applying the derivative operator a second time in Eq (13) and negating produces the observed Fisher information

$I:=-\frac{d^{2}}{d^{2}r}\ln L\left(\hat{r}\right)=\sum_{m=2}^{\tilde{M}} \left\{\mu\tilde{a}''_{m}\left(\hat{r}\right)+\tilde{\eta}_{m}\left[\left(\frac{\tilde{a}'_{m}\left(\hat{r}\right)}{\tilde{a}_{m}\left(\hat{r}\right)}\right)^{2}-\frac{\tilde{a}''_{m}\left(\hat{r}\right)}{\tilde{a}_{m}\left(\hat{r}\right)}\right]\right\}$,

so

$\hat{\sigma}^{2}\left(\ln\hat{r}\right)\approx\hat{r}^{-2}\hat{\sigma}^{2}\left(\hat{r}\right)\approx\hat{r}^{-2}I^{-1}=\frac{\hat{r}^{-2}}{\sum_{m=2}^{\tilde{M}} \left\{\mu\tilde{a}''_{m}\left(\hat{r}\right)+\tilde{\eta}_{m}\left[\left(\frac{\tilde{a}'_{m}\left(\hat{r}\right)}{\tilde{a}_{m}\left(\hat{r}\right)}\right)^{2}-\frac{\tilde{a}''_{m}\left(\hat{r}\right)}{\tilde{a}_{m}\left(\hat{r}\right)}\right]\right\}}$,

where the first approximation follows from the approximation , the second approximation is a standard use of Fisher information; and the final equality derives from Eq . Note that calculation of the expected folded AFS $\tilde{a}_{m}\left(r\right)$ ($m=2,3,...,\tilde{M}$) in Eq only requires $a_{m}\left(\hat{r}\right)$ for $m=2,3,...,M-2$.

The simplicity of Eq (19) suggests also comparing the approximate MLEs $\hat{r}^{\left(\Delta\right)}$ and $\hat{r}^{\left(I\right)}$ to an estimate $\hat{r}^{\left(M\right)}$ derived from applying the method of moments to $\tilde{\eta}_{-1}$. To begin, define

$h\left(r\right):=\mu\sum_{m=2}^{M-2} a_{m}^{\left(\Delta\right)}\left(r\right)$.

The moment

.

In a standard notation, let $h^{\circ-1}$ denote the inverse of a function $h$ under functional composition (often denoted by “”). In the method of moments, the basic reproduction number $\hat{r}^{\left(M\right)}\approx h^{\circ-1}\left(\tilde{\eta}_{-1}\right)$ is estimated from (the observable) $\tilde{\eta}_{-1}$. Logarithms $\ln r$ provide a convenient scale for $r>1$, so writing $\eta=\tilde{\eta}_{-1}$ temporarily (i.e., only to reduce visual noise in Eq , next), yields

.

Under the assumption that $\hat{r}^{\left(M\right)}\approx r$ (i.e., $\hat{r}^{\left(M\right)}$ has a small bias and coefficient of variation), the variance of Eq is

$\hat{\sigma}^{2}\left(\ln\hat{r}^{\left(M\right)}\right)\approx\frac{\sigma^{2}\left(\tilde{\eta}_{-1}\right)}{\left[\hat{r}^{\left(M\right)}h'\left(\hat{r}^{\left(M\right)}\right)\right]^{2}}\approx\frac{h\left(\hat{r}^{\left(M\right)}\right)}{\left[\hat{r}^{\left(M\right)}h'\left(\hat{r}^{\left(M\right)}\right)\right]^{2}}$,

because implies that .

Fig 2 for $\hat{r}^{\left(I\right)}$ has two analogs in this Appendix: Fig S1 for $\hat{r}^{\left(\Delta\right)}$ and Fig S2 for $\hat{r}^{\left(M\right)}$. The text surrounding Fig 2 explains the graphs for $\hat{r}^{\left(I\right)}$: Fig S1 and Fig S2 have the same structure as Fig 2.

**Fig S1. Plots for the maximum likelihood estimate (delta approximation)** $\hat{r}^{\left(\Delta\right)}$

Fig S1 for $\hat{r}^{\left(\Delta\right)}$ has the same format as Fig 2 for $\hat{r}^{\left(I\right)}$ in the main text.

Fig S1f is absent because for $\hat{r}^{\left(\Delta\right)}$ and $M=1024$, there were numerical difficulties associated with generating and multiplying extremely small infinite sums and extremely large binomial coefficients in Eq (4) for$a_{m}^{\left(\Delta\right)}$. Meticulous numerical programming might eliminate some of the numerical instability, but we judged the effort unjustified given the remarkable success of the estimator $\hat{r}^{\left(I\right)}$ .

**Fig S2. Plots for the estimate $\hat{r}^{\left(M\right)}$ from the method of moments**

Fig S2 for $\hat{r}^{\left(M\right)}$ has the same format as Fig 2 for $\hat{r}^{\left(I\right)}$ in the main text.

Fig S2 for $\hat{r}^{\left(M\right)}$ resembles Fig 2 for $\hat{r}^{\left(I\right)}$. It differs, however, in that near $\log_{10}r=0$, Fig S2e displays some systematic overestimation away from the perfect estimate $\hat{r}=r$, with Fig S2f showing an unacceptable worsening of the overestimation. The error estimate $\hat{\sigma}^{2}\left(\ln\hat{r}^{\left(M\right)}\right)$ in Fig S2d is slightly more accurate than $\hat{\sigma}^{2}\left(\ln\hat{r}^{\left(I\right)}\right)$ in Figure 2d, but the error estimate deteriorates badly in Fig S2f, presumably again from numerical difficulties caused by the infinite series.

The inferior estimators $\hat{r}^{\left(\Delta\right)}$ and $\hat{r}^{\left(M\right)}$ displayed a pronounced upward bias (of uncertain origin) in the important regime where $R_{0}>1$ is near 1.0 (see Fig S1e and Fig S2f). The two inferior estimators and their estimated errors displayed occasional numerical instabilities (for $\hat{r}^{\left(\Delta\right)}$, see Fig S1e, the non-existent Fig S1f; and for $\hat{r}^{\left(M\right)}$, see the large variations in $\hat{r}^{\left(M\right)}$ indicated by the dashed lines in Fig S2f). The inferior estimators can also involve staggeringly large binomial coefficients in computations that truncate infinite series. Current deep-sequencing technologies can generate 10^5^ reads of size comparable to gp120 [[1](#_ENREF_1),[2](#_ENREF_2)]. In applications utilizing extremely large sample sizes, therefore, the numerical extremes in $\hat{r}^{\left(\Delta\right)}$ and $\hat{r}^{\left(M\right)}$ render them undependable for routine computation.

# References

1. Carneiro MO, Russ C, Ross MG, Gabriel SB, Nusbaum C, et al. (2012) Pacific biosciences sequencing technology for genotyping and variation discovery in human data. BMC Genomics 13: 375.

2. McElroy K, Thomas T, Luciani F (2014) Deep sequencing of evolving pathogen populations: applications, errors, and bioinformatic solutions. Microbial Informatics and Experimentation 4: 1.
